# Resident Immune Cells of the Liver in the Tumor Microenvironment

**DOI:** 10.3389/fonc.2022.931995

**Published:** 2022-07-19

**Authors:** Yunjie Lu, Shiying Ma, Wei Ding, Pengcheng Sun, Qi Zhou, Yunfei Duan, Kurt Sartorius

**Affiliations:** ^1^ The Third Affiliated Hospital of Soochow University, Chanozhou, China; ^2^ Department of General Surgery, Wujin Hospital Affiliated to Jiangsu University, Changzhou, China; ^3^ Hepatitis Diversity Research Unit, School of Internal Medicine, University of the Witwatersrand, Johannesburg, South Africa; ^4^ Africa Hepatopancreatobiliary Cancer Consortium (AHPBCC), Mayo Clinic, Jacksonville, FL, United States; ^5^ University of Kwazulu-Natal Gastrointestinal Cancer Research Unit (UKZN/GICRC), Durban, South Africa

**Keywords:** immunology, tumorigenesis, adaptive, innate, cells, liver

## Abstract

The liver is a central immunomodulator that ensures a homeostatic balance between protection and immunotolerance. A hallmark of hepatocellular carcinoma (HCC) is the deregulation of this tightly controlled immunological network. Immune response in the liver involves a complex interplay between resident innate, innate, and adaptive immune cells. The immune response in the liver is modulated by its continuous exposure to toxic molecules and microorganisms that requires a degree of immune tolerance to protect normal tissue from damage. In HCC pathogenesis, immune cells must balance a dual role that includes the elimination of malignant cells, as well as the repair of damaged liver tissue to maintain homeostasis. Immune response in the innate and adaptive immune systems extends to the cross-talk and interaction involving immune-regulating non-hematopoietic cells, myeloid immune cells, and lymphoid immune cells. In this review, we discuss the different immune responses of resident immune cells in the tumor microenvironment. Current FDA-approved targeted therapies, including immunotherapy options, have produced modest results to date for the treatment of advanced HCC. Although immunotherapy therapy to date has demonstrated its potential efficacy, immune cell pathways need to be better understood. In this review article, we summarize the roles of specific resident immune cell subsets and their cross-talk subversion in HCC pathogenesis, with a view to identifying potential new biomarkers and therapy options.

## Introduction

The liver is a central player in immune regulation because of its constant exposure to gut-derived pathogens that require its multitude of innate and adaptive immune cells to respond to some pathogens and tolerate others. Immune response in the liver must carefully balance pro-inflammatory cytokines (IL-2/IL-7/IL-12/IL-15/IFN-γ) and anti-inflammatory cytokines (IL-10/IL-13/TGF-β) to coordinate resident and periphery leukocytes like T cells, B cells, macrophages (KCs/monocytes), natural killer (NK) cells, natural killer T (NKT) cells, and hepatic stellate cells (HSCs). Hepatocellular carcinoma (HCC), which accounts for approximately 90% of primary liver cancers, arises almost exclusively in a setting of chronic inflammation, which is a hallmark of HCC pathogenesis. This inflammation causes liver damage, compensatory tissue regeneration, and the activation of non-parenchymal cells that promote the development of fibrosis/cirrhosis. Over time, these conditions lead to chromosomal instability and the development of the tumor microenvironment (TME) (1).

The TME can be broadly classified into cellular and non-cellular components. The major cellular components include HSCs, fibroblasts, immune, and endothelial cells. These cell types produce the non-cellular components of the tumor stroma, including extracellular matrix (ECM) proteins, proteolytic enzymes, growth factors, and inflammatory cytokines. The non-cellular component of the tumor stroma regulates HCC pathogenesis by influencing cancer signaling pathways in the TME, tumor invasion, and metastasis (2). In these conditions, HCC pathogenesis promotes the constant expression of pro-inflammatory cytokines that outweigh the anti-inflammatory response to drive fibrogenesis resulting in the progressive buildup of fibrotic tissue, cirrhosis, and progression to HCC (3). Simultaneously, T-cell tolerance develops in the presence of a reduction in CD4^+^ effector T cells and an increase in T reg cells and T-cell exhaustion in the face of upregulated KCs and elevated IL-10/TGF-β (**Table 1**).

Despite the approval of a range of FDA-approved drugs for the treatment of HCC, the results have been disappointing and survival time has only been modestly extended. Innovative developments in immunotherapy, however, have improved the probability of developing a successful treatment for advanced HCC. In this review article, we summarize the roles of specific resident immune cell subsets and their pathways in HCC pathogenesis to outline potential new biomarkers of disease and therapy options.

## Resident Myeloid Immune Cells in the Liver

### Macrophages

Macrophages are an umbrella term for a diverse group of phagocytic cells (4). Hepatic macrophages are the first line of defense against pathogens, and this group of non-parenchymal cells consists mainly of resident and non-resident macrophages. Liver-resident macrophages that reside in the space of Disse consist of Kupffer cells (KCs) and monocytes that play a crucial role in chronic liver inflammation and the TME. Macrophages abundantly infiltrate the HCC microenvironment and tumor-associated macrophages (TAMs) promote tumorigenesis by regulating the immune responses to HCC cells and secreting various cytokines (5, 6) that promote ECM development and angiogenesis in tumorigenesis (7). Macrophages include two distinct polarization phenotypes, namely, M1 and M2 macrophages, that are triggered by their response to different microenvironments. M1 macrophages exert a cytotoxic function by releasing IL-1α, IL-1β, IL-12, IL-18, iNOS, and TNF-α, which are induced by LPS, IFN-γ, and GM-CSF. Conversely, M2 macrophages exert anti-inflammatory activities by expressing low levels of IL-12 and high levels of IL-10, arginase 1 and PD-L1, which are induced by IL-4, IL-10, IL-13, M-CSF, and helminth (5).

Monocyte-derived macrophages (MoMϕs) are derived from monocytes that are synthesized from hematopoietic stem cells (HSCs) in the bone marrow and enter the liver *via* the bloodstream (8). MoMϕs can also be classified as Ly-6C^high^ and Ly-6C^low^ monocytes. Ly-6C^high^ monocytes play an essential role in initiating HSC activation through secreting high levels of CCR2 (9) while the accumulation of Ly-6C^low^ monocytes attenuates liver fibrosis (10). Targeting CCR2 expression, therefore, has been demonstrated as a feasible therapeutic intervention (11). Interestingly, CD14^+^CD16^−^ monocytes in humans correlate with Ly6C^high^ monocytes in mice and are associated with Ly6C^low^ monocytes in mice (12).

Glucose homeostasis, which is mainly controlled by the liver, is vital for the energy demand of human organs and is partly mediated by SUV39H1, which makes it a potential target for therapy interventions (13). The importance of modulating glucose homeostasis is emphasized by its role in the promotion of inflammatory cytokines in macrophages (14).

**Figure 1:**
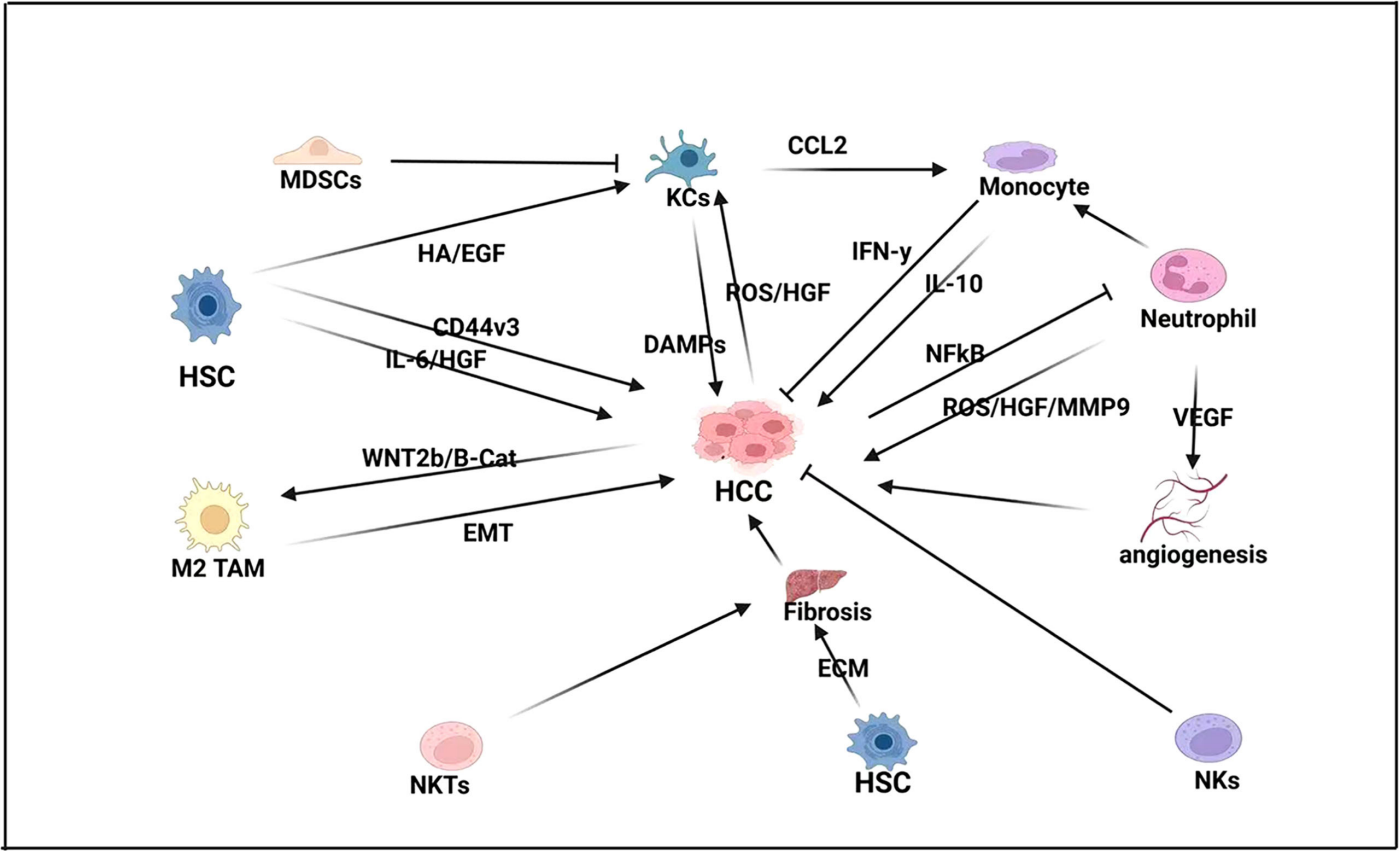
Innate immune response in HCC-BM. In HCC pathogenesis, hepatic stellate cells (HSCs) drive fibrogenesis by promoting extracellular matrix (ECM), as well as directly influencing proliferation by expressing pro-inflammatory cytokines like interleukin-6 (IL-6) and hepatocyte growth factor (HGF). HSCs also express the glycoprotein CD44v3 that promotes cell migration and invasion in HCC. Natural killer T (NKT) cells also promote fibrogenesis while neutrophils express vascular endothelial growth factor (VEGF) to promote angiogenesis and proliferation. Neutrophils also activate monocytes that can both repress HCC by expressing interferon-gamma (IFN-γ) and promote HCC by expressing interleukin-10 (IL-10). Kupffer cells (KCs) also play a crucial role in HCC pathogenesis by promoting monocytes by expressing C-C motive chemokine ligand-2 (CCL2), which acts as a monocyte attracting protein. KCs also express damage-associated molecular pattern molecules (DAMPs) and are activated by HSC. Expression of histamine (HA) and epidermal growth factor (EGF), as well as HCC expression of reactive oxygen species (ROS) and hepatocyte growth factor (HGF). However, KC expression is modulated by myeloid-derived suppressor cells (MDSCs).

Tumor-activated macrophages (TAMs) are associated with poor prognosis, as are two separate genes in TAM-like signatures, SLC40A1 and GPNMB (15). Therefore, TAMs that target the oncogenic expression of Wnt2b offer an exciting potential therapeutic strategy in HCC immunotherapy (16). In addition, Miz1 is a tumor suppressor that limits the ability of tumor hepatocytes to activate tumor-infiltrating macrophages and drive inflammation. Miz1 expression in HCC is negatively correlated with the phosphorylation of RelA and MTDH, as well as poorer overall survival and higher recurrence rates (17). Targeting the Miz1–MTDH–RelA axis, therefore, may also provide a potential therapeutic strategy for HCC. HOMER3-AS1 also drives HCC progression by regulating the behavior of tumor cells and macrophages, and HOMER3-AS1 may be another promising prognostic and therapeutic target for HCC (18) (**Figure 1**). Finally, TAM-elevated CCL2 levels are associated with reduced survival in HCC patients, thus demonstrating a new potential CCL2-targeted therapy for HCC (19).

Notch blockade impedes the differentiation of moTAMs but upregulates the Wnt/β-catenin signaling pathway to promote KclTAM proliferation and tumor-promoting cytokine production during hepatocarcinogenesis (20). CD74 is an independent predictor of HCC prognosis and CD74^+^ macrophages are closely associated with immunoreactive TME with CD8^+^ CTL function. Therefore, CD74 can be used as a predictive biomarker and potential therapeutic target for HCC (21).

### Kupffer Cells

KCs are tissue macrophages located in the lumen of the hepatic sinusoids. They are important members of the innate and adaptive immune systems. As antigen-presenting cells, KCs bridge the gap between the innate and adaptive immune systems. Following their activation by danger signals, KCs modulate inflammation and recruit immune cells, including large numbers of monocytes to the liver. KCs are a specific type of macrophage, and a combination of KCs and monocytes is involved in inflammation and wound healing by adjusting their phenotypes according to local signals (22). KCs in the liver act as sentinel cells to capture antigens and pathogens to maintain liver tolerance. Recruitment of macrophages is highly bactericidal and is a response to liver damage caused by acute inflammation, playing a key role in rapid infection control. Thus, the KCs that reside in the liver and the macrophages that they recruit are functionally different (23). Their constitutive ability reflects the potential of preserving tolerance of KCs in homeostasis by secreting IL-10, which can be stimulated in the presence of lipopolysaccharides (LPS). Under LPS challenges, IL-10, secreted by KCs plays a critical role in maintaining liver homeostasis (24), which involves a dynamic balance between inflammatory substances initiated by KCs and immune regulatory molecules. If this balance is broken, liver injury rises (25).

SNHG20 induces hepatic KC M2 polarization, through activation of STAT6, to promote the progression of NAFLD to HCC (26). Therefore, silencing SNHG20 expression could delay the progression of NAFLD to HCC. Upregulation of FTX inhibits the conversion of NAFLD to HCC by promoting M1 polarization of KC and provokes another effective target for the treatment of NAFLD-HCC (27). Taking an RNAi-based approach, microRNA-15a/16-1 attenuates immunosuppression by disrupting CCL22-mediated communication between KCs and Tregs, thus representing an additional potential immunotherapy approach for HCC (28).

### Dendritic Cells

DCs are antigen-presenting cells that not only participate in the intrinsic immune response but also serve as a bridge between intrinsic and adaptive immunity. DCs recognize and ingest exogenous antigens, present them to immature T cells and induce T-cell activation and proliferation. The unique DC compartment in the liver contains multiple DC subpopulations that secrete type I interferons, regulate NK cell activity, and induce antiviral immune responses (29). DCs also play an essential role in regulating the microenvironment by capturing and presenting antigens to activate effector cells in the immune system. Hepatic DCs also interact with CD4^+^ T cells during liver injury and tolerogenic DCs in the liver suppress the activation and proliferation of liver effective T cells to reduce the I/R injury (30). It has been demonstrated that a subset of human CD14^+^CTLA-4^+^ DCs can suppress CD4^+^ T cells by secreting IL-10 and IDO (indoleamine-2,3- dioxygenase) (31). DC-derived IDO is vital for the function of the immune regulating ability of liver-resident DCs and IFN-γ or low-level LPS is the main upstream activator that enhances the IDO signal (32). Importantly, IL-6-mediated STAT3 activation is necessary for the DC differentiation of IDO-producing regulatory DCs in the liver. In addition, foreign DNA and HMGB1 immune complexes also activate liver-resident DCs *via* interacting with the receptors of TLR9 and RAGE (33).

Hypoxia-inducible factor 1-α (HIF-1α) transcriptionally upregulates the expression of the ectonucleotidases CD39 and CD73 in HCC cells and induces the production of extracellular adenosine (eADO) that significantly promotes pDC recruitment into tumors *via* the adenosine A1 receptor (ADORA1). High-density tumor-infiltrating pDCs are associated with poor prognosis in HCC patients offering a potential role for targeting pDC recruitment as a potential adjuvant immunotherapy for HCC (34). Intratumor pDCs may promote HCC progression and recurrence through induction of immune tolerance by Treg cells and an inflammatory TME of IL-17^+^ cells. Targeting anti-immune responses induced by TA-pDCs, therefore, may become an additional new strategy for the clinical treatment of HCC (35). In addition, CD40L co-stimulation could also provide a promising tool for enhancing DC immunotherapy in liver cancer (36).

## Liver Lymphoid Immune Cell Populations

### T Cells

**Figure 2:**
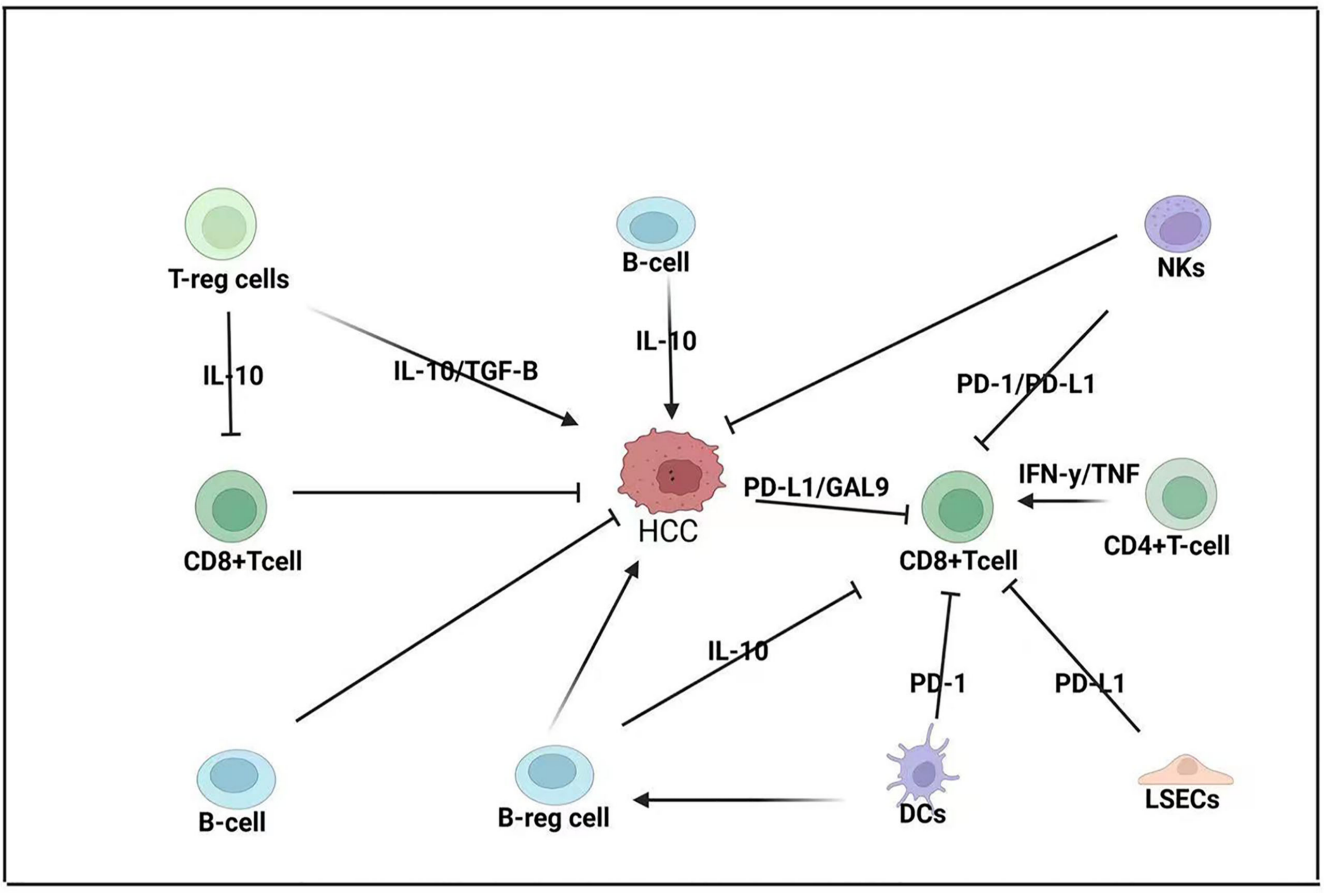
Adaptive immune response in HCC-BM. CD8^+^ T cells are considered the primary anti-cancer cells in HCC pathogenesis but their function is modulated by both the TME and the innate immune system. CD8^+^ T cells are modulated by regulatory T cells (T-reg cells) that express interleukin 10 (IL-10); T-reg cells can also promote the expression of transforming growth factor beta (TGF-B), which promotes HCC. Regulatory B cells (B-reg cells) can also directly promote HCC by expressing IL-10 or modulating CD8^+^ T-cell expression. HCC tumors can express programmed death ligand-1 (PD-L1) and galactin-9 (GAL-9) to repress CD8^+^ T cells that can also be repressed by liver sinusoidal endothelial cells (LSECs) that can express PD-L1 and dendritic cells (DCs) that express programmed cell death protein 1 (PD-1). Natural killer (NK) cells can repress both CD8^+^ T cells and HCC pathogenesis.

T cells occupy a central position in the adaptive immune response. Mature T cells are divided into CD4^+^ T cells and CD8^+^ T cells, which are mainly involved in the transduction of T-cell activation signals (**Figure 2**). CD4^+^ T cells and CD8^+^ T cells are crucial mediators of the intrahepatic antiviral immune response. Liver parenchymal and non-parenchymal cells protect against the tolerogenic environment by suppressing T-cell responses (37, 38). This mediation commonly leads to the balance between immune response and tolerance under steady-state conditions. Liver injury mediated signals, though, may override the tolerance and induce immune effector cellular activation. Upregulation of ICAM-1, VCAM-1, and VAP-1 and other adhesion molecules are crucial factors that induce the activation of T cells (39–42). Interestingly, TGF-β is essential for the survival and development of T-cell subsets and could be reverted by silencing SUV39H1. SUV39H1 inhibits TCR-mediated IL-2 transcription *via* the TGF-β-Smad pathway. Knockdown of SUV39H1 partially blocked TGF-β-mediated IL-2 inhibition; thus, SUV39H1 may become a new target for autoimmune disease therapy (43). SUV39H1-deficient Th2 cells express Th1 characteristic genes, and intervention of SUV39H1 may have a therapeutic effect in Th2 cell-mediated inflammatory diseases (44).

In another study, it was demonstrated that norisoboldine downregulated the glycolytic process of CD4^+^ T cells under hypoxic conditions, reduced NAD^+^ and SIRT1 levels, promoted ubiquitin-proteasomal degradation of SUV39H1 protein, and inhibited the enrichment of H3K9me3 in the Foxp3 promoter region of CD4^+^ T cells, thereby enhancing Treg polarization (45). Since Th1, Th2, and Treg cells are essential for liver immunology, the SUV39H1 network needs to be further investigated.

The relationship between the liver and Treg cells under various pathophysiological conditions is being gradually revealed in recent years. KCs can induce Treg cells *via* the secretion of prostaglandins (PG) E2 and 15d-PGJ2 (46, 47). In another study, pre-treatment of KCs by IFN-γ indicated an upregulation of the enzyme IDO *in vitro* (48). Under pathological conditions, the strong immunosuppressive ability of Treg can control liver inflammation, reduce liver damage, and regulate immune tolerance during liver transplantation. However, the Treg cell’s ability to suppress the immune response may also lead to chronic viral hepatitis and speed up the growth of tumors by helping the tumors evade immune response (49).

### Natural Killer T Cells

NKT cells are a particular subset of T cells with both T-cell receptors (TCRs) and NK receptors on the cell surface. NKT cells can produce a large number of cytokines and induce the same cytotoxic effect as NK cells. Liver sinusoidal endothelial cells (LSECs) and KCs can secrete CXCL6 that promotes the homing of CXCR6^+^ NKT cells (50, 51). Liver dendritic cells (DCs) also interact and activate patrolling NKT cells by expressing IL-4 and IFN-γ through IL-12 (52, 53).

The nuclear factor TOX promotes CD8^+^ T-cell depletion in HCC by regulating the intracellular recycling of PD-1. Downregulation of TOX expression in CD8^+^ T cells has a synergistic effect with anti-PD-1 treatment. Thus, TOX may be a promising target for reversing T-cell depletion and enhancing anti-tumor immunity (54). Moreover, 4-1BB co-stimulation further enhances T-cell activation after PD-1 blockade, and immunotherapy targeting co-stimulatory receptor 4-1BB, in combination with anti-PD-1 therapy, may be an effective therapeutic strategy (55).

### Innate Lymphoid Cells

ILCs are vital in maintaining metabolic balance and act as an anti-infection immune response. The ILC family includes ILC1, which mainly secretes IFN-γ; ILC2, which primarily secretes IL-5, IL-9, and IL-13; and ILC3, which mainly expresses IL-22 and IL-17 (56). In the liver, ILC1 reduces the severity of acute liver injury by regulating Bcl-xL expression by hepatocytes (33) while ILC2 controls liver fibrosis and tissue repair by interacting with macrophages following injury (57, 58). A human experiment study showed that ILC3 promoted fibrosis by expressing IL-17 and IL-22 (59) and that the tumor cytokine microenvironment controls the composition of ILC and the prognosis of HCC. In addition, patients with a high ILC2/ILC1 ratio that express IL-33 in their tumors promote the production of ILC2 and induce an increase in CD8^+^ T cells and a decrease in regulatory T cells (Treg) in tumors, resulting in improved patient survival. Thus, modulation of the cytokine gradient of ILC may enhance the antitumor immune response in HCC (60). In other studies, ILC3, which lacks the natural cytotoxic trigger receptor (NCR-ILC3), promotes HCC development in response to interleukin 23 (IL-23). Furthermore, NCR-ILC3 initiates IL-17 production in response to IL-23 stimulation and directly suppresses CD8^+^ T-cell immunity by promoting lymphocyte apoptosis and limiting their proliferation (61). Thus, NCR-ILC3 should also be considered a target for future tumor immunotherapy.

### Natural Killer Cells

NK cells are essential liver-resident lymphocytes in the innate immune system. The phenotypes/functions of NK cells in the liver are different from those of peripheral circulation NK cells, containing subsets with tissue-resident characteristics to resist viral infection and tumor immune surveillance. NK cell phenotypes are altered and dysfunctional in the disease environment, indicating that NK cells are essential in mediating the immunology of the liver under steady-state conditions. Liver-resident NK (LR-NK) cells also play a crucial role in liver immune homeostasis maintenance. LR-NK cells co-locate with CD4^+^ T cells and can significantly suppress T-cell proliferation and function. In contrast, NK cells from the circulation system do not have this ability (62). Recently published data also indicated that liver-resident NK cells suppressed T cells’ antiviral ability through controlling PD-1/PD-L1 signaling (63).

Multiple studies have investigated various signaling pathways influencing NK expression. SIRT2, for instance, may enhance the tumor-killing effect of NK cells by activating the ERK1/2 and p38MAPK signaling pathways and is potentially a new therapeutic target for immunotherapy of liver cancer (64). In another study, Glypian-3 (GPC3) was shown to be a suitable target for Chimeric antigen receptor (CAR) therapy in HCC, and therapeutic approaches using GPC3-targeted antibodies or peptide vaccines are safe (65). Patients with high CD96 expression in tumors have a poor clinical prognosis, and the blockade of CD96–CD155 interaction or TGF-β1 restores NK cell antitumor immunity by reversing NK cell depletion (66). Interestingly, the expression of failure-associated checkpoint molecules such as PD-1, CD96, and TIGIT on CD49a^+^ NK cells within the tumor is upregulated, allowing tumor cells to escape from immune surveillance, and CD49a^+^ NK cell accumulation in liver tumor tissue is associated with disease progression and poor prognosis (67). In addition, blocking the inhibitory receptors NKG2A, TIGIT, LAG3, or KIR, expressed on NK cells with antibodies, is a potential therapeutic strategy (68). Finally, micro-RNA like miR-561-5p promotes HCC metastasis by inhibiting its target CX_3_CL1, thereby blocking NK cell recruitment and infiltration. One study demonstrated that miR-561-5p/CX_3_CL1/CX_3_CR1^+^ components of the NK cell axis are potential immunotherapeutic targets in HCC (69).

### B Cells

B cells not only mediate humoral immune responses through antibody production but also present antigens and participate in immune regulation. However, the role of tumor-infiltrating B cells (TIBs) remains controversial. Experimental data using mice deficient for B cells [Igh6(-/-), μMT] and data on mRNA expression in human HCC suggest that T cells prevent initial tumor formation, while B cells critically limit the growth of established tumors (70). In addition, the number of TIBs in HCC correlates with T cells and tumor-infiltrating T- and B-cell density correlates with high survival rates in HCC (71). The density of TIBs also correlates with the activation of bothCD8^+^ T and CD56^+^ NK cells intratumorally, which may lead to an enhanced local antitumor immune response (71). The critical role of T-cell and B-cell interactions in cancer progression provides new ideas for immunotherapy. In particular, CD40 is a co-stimulatory molecule expressed on B cells that activates T cells and B cells when linked to CD40 ligands, making CD40 a promising target for immunotherapy (1).

B-cell-mediated IL-10 expression is known to suppress CD4^+^ T cell proinflammatory cytokine expressions, such as TNF-α, IFN-γ, and IL-17; inhibit CD8^+^ T-cell cytotoxicity; and promote Treg cell differentiation (72). The increase of IL-10-expressing B cells could contribute to the immune inhibition in the liver microenvironment (73) and TLR-2 and TLR-9 agonists were shown to increase IL-10 production in B cells (74). In addition, the presence of IgA^+^ plasma cells expressing PD-L1 and IL-10 in the tumor environment was associated with poor T-cell immunity in human liver cancers (75). Recently, it was found that GABA secreted by B cells promotes the differentiation of monocytes into anti-inflammatory macrophages, which secrete IL-10 and suppress the anti-tumor response of CD8^+^ T cells (76).

## Immune-Regulating Liver Non-Hematopoietic Cells

### Liver Sinusoidal Endothelial Cells

In the liver, up to 50% of the non-parenchymal cells are LSECs. LSECs are interrelated with the apoptosis of activated T cells and affect the function of DCs in several ways. LSECs are located in an ideal anatomical position that can absorb exogenous antigens efficiently and rapidly and their expression eventually promotes immune tolerance to a specific antigen (77). The space of Disse is a narrow space of approximately 0.4 μm between LSECs and hepatocytes, which is in continuity with the sinusoidal lumen. Stellate cells are found in the space of Disse, while KCs and intrahepatic lymphocytes are arranged in the lumen of the hepatic sinusoids. The slow blood flow in the hepatic sinusoids and the unique structure of LSECs facilitate prolonged contact between lymphocytes and antigen-presenting cells and promote lymphocyte extravasation. In addition, hepatocytes and LSECs secrete the chemokine CXCL9 to recruit lymphocytes into the liver (78). Similar to DCs, LSECs are crucial in receptor-mediated endocytosis and/or phagocytosis, antigen processing and presentation (78). Human LSECs express ICAM-1, TNF α and IFN-γ, and induce high expression of MHC-II, CD40, ICAM-1, and VCAM-1. LSECs can also activate the interaction between hepatic sinusoidal endothelial cells and immune cells. Under normal circumstances, hepatic sinusoidal endothelial cells will trigger an anti-inflammatory response after antigen presentation to create a self-balanced environment. KLF2-NO signaling in LSECs can also inhibit tumor progression by inducing CXCL16 overexpression and recruiting NKT cells (79). Finally, the overexpression of PD-L1 by LSECs inhibits CD8+ T-cell activation, leading to a poor prognosis of HCC (77).

### Hepatocytes

Hepatocytes, which express a wide array of innate immune receptors, account for 90% of all liver cells. With the immune receptors, they play an irreplaceable role in metabolism, protein production, toxin neutralization, pathogen detection, and the host immune response. Hepatocytes express toll-like receptors (TLRs) that respond to TLR2 and TLR4 ligands (80), as well as act as antigen-presenting cells to naive T cells by physically interacting in an ICAM-1/MHC-dependent pathway (81). Hepatocytes are responsible for the production of most of the acute phase proteins and their complement components that are the first line of defense against pathogens (82).

AXIN1 is a negative regulator of the Wnt/b-linked protein signaling pathway. AXIN1 mutant HCC induces the Notch and YAP pathways, and these pathways also provide new therapeutic targets for AXIN1 mutant HCC (83). Finally, siRNA-mediated transient GPC3 silencing inhibits the invasion and migration of hepatocellular carcinoma cells. Therefore, GPC3 can be investigated as an immunotherapeutic target for HCC (84).

### Hepatic Stellate Cells

HSCs exist in the space of Disse and account for 30% of non-parenchymal cells. In normal liver conditions, HSCs are at rest and have a low ability to synthesize collagen and their primary function is to store retinoids. TLR4 and TLR9 are expressed in HSCs (85) and TLR4 directly stimulates HSCs to secrete chemokines (CCL2, CCL3, and CCL4), thus exhibiting pro-inflammatory features. Interestingly, TLR9 signaling enhances collagen production in HSCs but inhibits HSC migration to regulate liver fibrosis. In addition, a series of related transcription factors may be involved in the formation of liver fibrosis through different mechanisms (86, 87). For example, the c-Abl-MRTF-A positive feedback loop contributes to HSC activation and liver fibrosis, and MKL1 interacts with AP-1 and SMAD3 to activate CTGF transcription to promote HSC activation in a non-autonomous fashion (88, 89).

GDF15 is an important mediator in the TME linking HSCs and hepatic tumor cells, to promote the progression of HCC. Therefore, the anti-GDF15 neutralizing antibody may be a novel therapeutic agent for patients with HCC (90). HSC-induced N-methyltransferase (NNMT) promotes HCC cell invasion and tumor metastasis by enhancing the expression of CD44v3, making it a promising prognostic biomarker and therapeutic target for HCC (91). HSCs promote MDSC migration *via* the SDF-1/CXCR4 axis, thereby promoting tumor progression (92). In this regard, the miR-1246-RORα-Wnt/β-catenin axis is a novel pathway for HSCs to promote HCC progression, and thus miR-1246 and RORα may become new therapeutic targets for HCC (93). The Sox9/INHBB axis also promotes the growth and metastasis of HCC tumors *in situ* by activating HSCs in the TME, which may also be a potential target for HCC therapy (94).

## Cross-Talk Between Innate and Adaptive Immune Systems

In HCC pathogenesis, immune cells in both pathways are both activated and repressed (see **Table 1**). In HCC progression leading to metastasis, the homeostatic cross-talk between the innate and adaptive immune system is dysregulated in an increasing manner (4). In general, CD8^+^ T cells are often considered to be the main anti-cancer immune cells (95) and a common feature is the subversion of their priming, which occurs in three ways. This occurs *via* the repression of TCR interaction with MHC class 1 peptides, the absence of co-stimulation of other receptors (CD28), and the repression of cytokine signals such as IL-12 IFN-1 (96).

**Table 1:** Expression of innate and adaptive immune cells.

Immune cells	Expression in HCC
CD8^+^ T cells CD4^+^ effector T cells Treg cells B cells KCs or monocytes NKT cells NK cells HSCs DCs Neutrophils	Dysregulated up/down Dysregulated up/down Upregulated Dysregulated up/down Upregulated Downregulated Downregulated Upregulated Upregulated Upregulated

Immune escape is also facilitated by dysfunction or a reduced number of DCs that facilitate CD8^+^ T-cell priming (97). In addition, NK interaction with DCs (cDC1) is essential to promote their effectiveness and abundance (98). In the TME, the stimulation of CCL2 often promotes the abundance of monocytes that can act as a barrier to intratumoral penetration of antigen-specific T cells that gather in the surrounding stroma (99). Myeloid-derived suppressor cells (MDSCs) consist of monocytes and neutrophils. In carcinogenesis, a wide range of cancer-induced monocytes repress CD8^+^ T cells and IFN-γ (100), as well as promote Tregs to promote tumor growth. Interestingly, monocytes can also promote NKs (101). Treg stimulation also occurs *via* TAM-stimulated CCL2 (102), as well as *via* TAM expression of PD-L1/2 to repress TCR/CTLs to promote a strong immunosuppressive effect to illustrate the cross-talk between TAMs and the adaptive immune system (103). In another example of cross-talk, CD8^+^ T cells can also be repressed by neutrophils in the metastasis stage (104). Treg expression that is promoted by DCs (105) can also repress CTLs and promote NK apoptosis (106, 107). Finally, Breg expression that is promoted by B-cell production of IL-10 (108) can promote CD4^+^ T-cell stimulation of FOXP3 Tregs (109).

## Discussion

Immune response in the liver incorporates a network of lymphocytes to both respond to pathogens and injury, and maintain homeostasis. Dysfunctional inflammatory mechanisms may lead to liver injury, non-resolving hepatitis, and eventually carcinogenesis. The function of hematopoietic progenitor cells may decrease this inflammatory activity and maintain liver homeostasis to protect from tissue damage and reduce infection and metastasis. Many other studies have defined the characteristics, function, and mechanism of several other organ-specific immune cells including skin, lung, and other tissue; however, limited attention has been focused on the liver’s residential immune cells. Further studies, using new technologies such as single-cell sequencing and mass spectrometry streaming, may contribute to a more profound understanding of liver-specific immunology to maintain homeostasis, as well as respond to carcinogenesis. The limited efficacy of immunotherapy to date for the treatment of HCC requires the investigation of new immunogenic targets.

## Author Contributions

All authors listed have made a substantial, direct, and intellectual contribution to the work, and approved it for publication.

## Funding

This work was supported by the National Natural Science Foundation of China (Grant No. 81971504), Post-Doctoral Special Foundation of China (2020M670065ZX), Post-Doctoral Foundation of Jiangsu Province (Grant No. 2020Z021), Changzhou Social Development Foundation (CE20205038), Changzhou Science and Technology Planning Project (CE20215042), Jiangsu 333 Talent Training Project (2022 3-4-116), Changzhou Health Commission Major Science and Technology Project (ZD201901), Young Talent Development Plan of Changzhou Health Commission (CZQM2020118), the Development Foundation of Affiliated Hospital of Xuzhou Medical University (XYFY2020016), DAAD-K.C. WONG FUNDING (57501535) and the Changzhou Sci&Tech Program (CJ20210013, CJ20220008).

## Conflict of Interest

The authors declare that the research was conducted in the absence of any commercial or financial relationships that could be construed as a potential conflict of interest.

## Publisher’s Note

All claims expressed in this article are solely those of the authors and do not necessarily represent those of their affiliated organizations, or those of the publisher, the editors and the reviewers. Any product that may be evaluated in this article, or claim that may be made by its manufacturer, is not guaranteed or endorsed by the publisher.
